# Three-Dimensional Printing of Graphene Oxide/Poly-L-Lactic Acid Scaffolds Using Fischer–Koch Modeling

**DOI:** 10.3390/polym15214213

**Published:** 2023-10-25

**Authors:** Thamires Santos da Silva, Bianca de Oliveira Horvath-Pereira, Leandro Norberto da Silva-Júnior, João Víctor Barbosa Tenório Fireman, Michel Mattar, Marcílio Félix, Rogerio Leone Buchaim, Ana Claudia Oliveira Carreira, Maria Angelica Miglino, Marcelo Melo Soares

**Affiliations:** 1 Departament of Surgery, School of Veterinary Medicine and Animal Science, University of São Paulo, São Paulo 05508-270, SP, Brazil; thamiresssilva@usp.br (T.S.d.S.); horvath@usp.br (B.d.O.H.-P.); silvajunior@usp.br (L.N.d.S.-J.); jfireman9@alumni.usp.br (J.V.B.T.F.); aconc.iq@usp.br (A.C.O.C.); miglino@usp.br (M.A.M.); 2 Instituto de Reabilitação Oro Facial Osteogenesis S/S LTDA, Vila Olimpia 04532-060, SP, Brazil; michelmatar@gmail.com; 3 Department of Animal Anatomy, University of Marilia, Mirante, Marília 17525-902, SP, Brazil; felix.marcilio@hotmail.com; 4 Department of Biological Sciences, Bauru School of Dentistry, University of São Paulo, Bauru 17012-901, SP, Brazil; rogerio@fob.usp.br; 5 Center for Natural and Human Sciences, Federal University of ABC, Santo André 09210-580, SP, Brazil

**Keywords:** bone tissue, spectroscopy, trabecula, biocompatibility

## Abstract

Accurately printing customizable scaffolds is a challenging task because of the complexity of bone tissue composition, organization, and mechanical behavior. Graphene oxide (GO) and poly-L-lactic acid (PLLA) have drawn attention in the field of bone regeneration. However, as far as we know, the Fischer–Koch model of the GO/PLLA association for three-dimensional (3D) printing was not previously reported. This study characterizes the properties of GO/PLLA-printed scaffolds in order to achieve reproducibility of the trabecula, from virtual planning to the printed piece, as well as its response to a cell viability assay. Fourier-transform infrared and Raman spectroscopy were performed to evaluate the physicochemical properties of the nanocomposites. Cellular adhesion, proliferation, and growth on the nanocomposites were evaluated using scanning electron microscopy. Cell viability tests revealed no significant differences among different trabeculae and cell types, indicating that these nanocomposites were not cytotoxic. The Fischer Koch modeling yielded satisfactory results and can thus be used in studies directed at diverse medical applications, including bone tissue engineering and implants.

## 1. Introduction

Bone defects caused by trauma, pathologies, or infections are among the most common problems in medical clinics [1,2,3,4,5]. Critical bone defects cannot self-repair and require surgical intervention [3,6,7]; however, the reconstruction of these deformities is challenging. Among clinical treatment options, autograft is considered the gold standard, despite its limitations due to low availability of bone substitutes, donor site morbidity, potential pathogen transmission, and functional impairment [8,9].

To minimize such problems, bone tissue engineering (BTE) has evolved to offer alternative solutions for the development of biological substitutes and the improvement of tissue regeneration through printing [3,10]. However, accurately printing customizable scaffolds with relevant properties (e.g., size, shape, and structural integrity) that achieve optimal results in regeneration is challenging because of the complexity of bone tissue composition, organization, and mechanical behavior [10,11].

Significant advances have been made in 3D printing technology for BTE by combining biomaterials that provide physical and mechanical support to produce scaffolds that mimic the macro- and microstructure of the target tissue [3,12,13,14]. Tavakoli et al. [15] in their studies, combined GO and PLLA to evaluate the combination’s potential for the use in bone regeneration. The authors demonstrated that the combination of these nanocomposites was highly promising by presenting an excellent scaffold bioactivity, cellular adhesion, and proliferation, besides the differentiation of the bone marrow mesenchymal stem cells (CTMs). These features are important in areas exposed to high mechanical stress, where strength and stability are essential to provide structural and functional support, such as in the mandible [16,17].

Microscopically, the mandible is characterized by two bone tissues: the cortical tissue (outer layer), which has a dense and compact structure, composed of structural units called osteons, and the medullar tissue (inner layer) composed of a trabecular structure forming a three-dimensional network of interconnected trabeculae or bone laminae [18,19]. Knowing these structures is important for modeling from a structural perspective, as the material produced must have adequate porosity, interconnectivity, and size to allow proper cell nutrition, nutrient exchange, migration, and cell adhesion [3].

CAD/CAM (computer-aided design/computer-aided manufacturing) technology allows the defect to be studied through modeling and individualization, replicating the region of defect to be repaired on a macro- and microscales [20]. Once the virtual scaffold is obtained in the CAD-CAM program, it is printed by layer-by-layer stacking, following the designed 3D model, with high precision and customization [10,21,22]. 

Poly-L-lactic acid (PLLA) is among the materials that have attracted attention in bone replacement due to its degradability by hydrolysis, as well as its good biocompatibility and processability [5,23,24]. On the other hand, PLLA has low mechanical properties (strength = 60 Mpa, elasticity = 220 Mpa), is hydrophobic, and lacks osteogenic activity [9]. To compensate for these disadvantages, an alternative approach is to combine PLLA with graphene oxide (GO). This compound offers several advantages, including a high surface area, excellent hydrophilicity, good mechanical properties, and the capability to promote cell proliferation and differentiation [25,26,27,28,29].

Although the use of both nanocomposites in bone regeneration has already been described [15,28,30], printing both materials in combination using the Fischer–Koch modeling has not yet been studied. This geometry is a model studied in triple periodic minimal surfaces (TPMSs), which is a mathematically defined structure that repeats in three dimensions with a zero medium curvature [31]. Besides this, due to its internal interconnected porous structure, strength, and relatively high mechanical energy absorption, it can also be adjusted to achieve parameters such as pore size, porosity, shape, and permeability favorable for the BTE scaffolds [32].

Therefore, this study characterized the properties of PLLA/GO-printed scaffolds using Fourier-transform infrared (FTIR) spectroscopy, Raman spectroscopy, and scanning electron microscopy (SEM). We aimed to achieve the reproducibility of the trabecula planned in a virtual environment onto a printed piece, as well as to assess its response to the cell viability assay. 

## 2. Materials and Methods

### 2.1. Ethics Committee

The present study was approved by the Committee for Ethics in the Use of Animals (CEUA) at the Faculty of Veterinary Medicine and Animal Science of the University of São Paulo.

### 2.2. GO/PLLA Nanocomposite Production

GO powder was produced by chemical exfoliation of graphite (Nacional de Grafite Ltd.a^®^, São Paulo, Brazil) following the modified Hummer’s method [33]. Then, 0.2% (wt) of GO was added to the polymer (Evonick RESOMER^®^ L 210 S). The mixture was extruded using an extruder standard screw (Thermo Fisher Scientific, Karlsruhe, Germany, Process L/D 40 n° 11). The extruded filaments were cooled on a ventilated belt with controlled winding tension to obtain 1.75 mm thick filaments. This procedure was conducted at the Mackgrape laboratory at Mackenzie Presbyterian University, São Paulo, Brazil. The filaments were subsequently stored in a dehumidifier until printing, as described in a previous study [28].

### 2.3. Trabecular Modeling

First, the 3D-printed trabecula and its response to cell adherence were tested. For this, the test specimens were modeled according to the Fischer–Koch standard [34], because, among the geometries that most resembles the bone trabeculate, the Fischer–Koch is the one that presents a better density/mechanical resistance ratio and better fluidity [35], with trabeculae ranging from 200 to 600 mm (spacings 400 a 5000 mm, line width 200 mm, layer thickness 200 mm).

**Figure 1:**
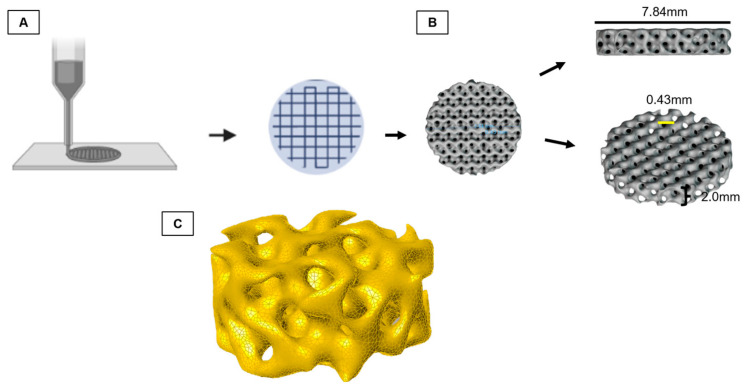
Schematic diagram of graphene oxide/poly-L-lactic acid (GO/PLLA) test specimen printing. (**A**) Layer by layer printing; (**B**) trabecula dimensions; (**C**) Fischer–Koch computational model of the trabecula.

Next, an STL (standard triangle language) file was produced (Figure 1). The file was printed by fused deposition modeling (FDM) on an Ender 3 printer (Creality 3D^®^) using the Creality Print (V4.3.5.5769) software and printing settings for commercial PLLA. The printing produced the test specimens (7.84 × 2.00 × 0.43 mm) that were sterilized through autoclaving (BIOEX-ABHD, 21L, 121 °C/ 30 min, and 1 kgf/cm^2^ of pressure). Moreover, the test specimens were subjected to sterility tests (incubated in MEM/Alpha-LGCBio medium at 37 °C) for 72 h to check for potential contamination.

### 2.4. GO/PLLA Scaffold Printing for Critical Defect in Goat

A Digital Imaging and Communications in Medicine (DICOM) file was obtained from tomography (GE ACTS 16/32, GE Healthcare, Chicago, IL, USA) with a 0.5 mm resolution, using volumetric scanning, FOV for specific region, 6 mm slice thickness, image matrix of 512 × 512 pixels of the goat mandibular bone. This bone was chosen because of its trabecular microstructure, which is similar to that of the human mandible, and its suitability for the biomechanical testing of implants [36]. The DICOM file was then converted to a standard triangle language (STL) file and used to plan the osteotomy and induce a critical defect in the mandibular angle (between the body and ramus of the mandible) in the DDS Surgery program (JST sp. z. 0.0. ul. Wały Dwernickiego 43/45 42-200 Częstochowa, Poland-CE 0197). Subsequently, a new STL file of the mandible was generated with the dimensions of the scaffold (4.5 cm × 3.0 cm). This file was then exported to the Blender software (2.79b version, Blender^®^) for modeling the osteotomy guide. When modeling the scaffold, the trabecula test specimen (7.84 × 2.00 × 0.43 mm) was produced for in vitro testing. 

**Figure 2:**
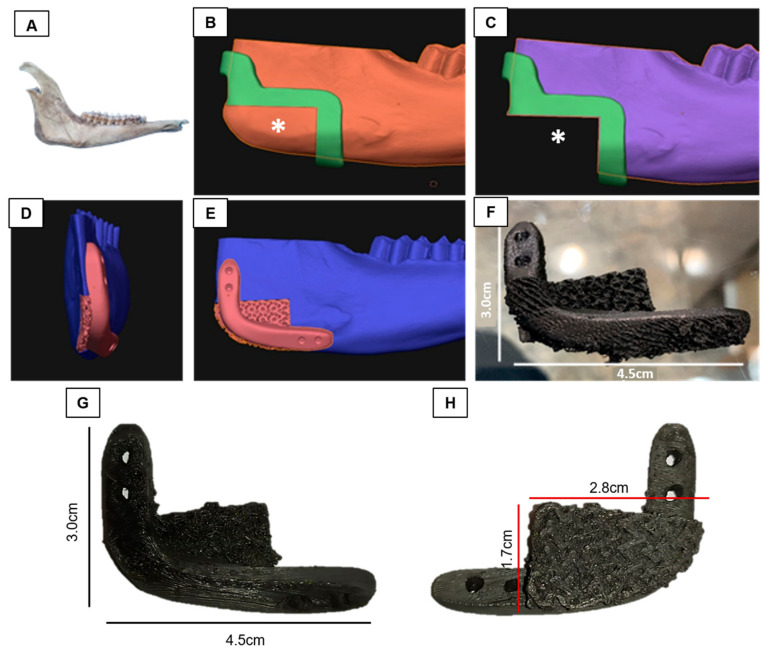
Production of graphene oxide/poly-L-lactic acid (GO/PLLA) scaffolds with critical defect. (**A**) Goat mandible; (**B**,**C**) implant site (*); (**D**,**E**) printing model of the fixation site; (**F**) printed scaffold; (**G**) lateral view of the printed scaffold; (**H**) medial view of the printed scaffold.

To secure the critical defect scaffold in place, a reinforced bar with an extension for attaching four screws was added. The scaffold’s surface was designed to have a similar texture to the bone trabeculate in the printing. However, the first layer, to avoid the deposited cells during culture to surpass the scaffold before the cell’s adhesion, was printed in a less spaced manner. The files were then transferred to an FDM printer for slicing and modeling for printing. The osteotomy guides were printed in ABS (acrylonitrile butadiene styrene) for the final touch (Figure 2). Subsequently, the scaffolds were subjected to sterility tests incubated in MEM/Alpha culture medium (LGCBio, Cotia, São Paulo, Brazil) at 37 °C for 72 h to check for potential contamination.

### 2.5. Fourier-Transform Infrared (FTIR) Spectroscopy Analysis

The resomer (PLLA) and nanocomposites (GO/PLLA) were qualitatively characterized for their chemical composition using FTIR spectroscopy. This analysis was conducted using the IRAffinity-1S spectrometer (Shimadzu, Tokyo, Japan) and the attenuated total reflectance (ATR) accessory, with a zinc selenide (ZnSe) crystal at room temperature. Measurements were taken in the wavenumber range of 4000 to 500 cm^−1^, and at least 20 readings at 4 cm^−^^1^ resolution were taken for each sample. For analysis of the powdered GO, it was previously compacted.

### 2.6. Raman Spectroscopy Analysis

Raman spectroscopy was also used to detect physicochemical changes in the analyzed materials. In this way, the spectra of the GO, PLLA and GO/PLLA nanocomposites were obtained using the Witec UHTS 300 Raman spectrophotometer coupled to an optical microscope. For this analysis, the excitation laser with a wavelength of 532 nm was used.

### 2.7. Cell Adhesion Test of Test Specimens and Critical Defect Scaffolds

Test specimens (*n* = 18) and critical defect scaffolds (*n* = 12) were washed with PBS supplemented with 1% antibiotic (penicillin-streptomycin, LGC Biotechnology, Cotia, São Paulo, Brazil) for 5 min, placed on 35 mm plates, washed with 70% EtOH alcohol, exposed to ultraviolet (UV) light for 15 min, and washed again three times with PBS. Then, 2 × 10^5^ goat mesenchymal cells were used. The cells were grown using α-MEM culture medium (LGC Bio, Cotia, São Paulo, Brazil), supplemented with 10% fetal bovine serum (FBS) and 1% antibiotic, for 7 days at 37 °C and 5% CO_2_. Medium changes were performed every two days. Next, the samples were fixed in 4% paraformaldehyde for 24 h for SEM analysis.

### 2.8. Study of Three-Dimensional Architecture and Cell Adhesion in Test Specimens and Critical Defect Scaffolds

SEM analyses were conducted to obtain a three-dimensional view of the scaffold printing and analyze the architecture of the scaffolds (test piece and critical defect). The structure of the critical defect scaffolds was evaluated, along with any similarities to the test specimen and the anatomy of the mandibular angle in the goat species.

To evaluate cell adhesion after 7 days of culturing with goat mesenchymal cells, both the critical defect scaffold and test specimen were fixed in a 4% PFA solution diluted in phosphate buffer for 24 h. Subsequently, they were dehydrated in a series of increasing alcohol concentrations (70–100%) for 10 min each and mounted on stubs using double-sided carbon tape. In the metallization step, a layer of gold (≈20–30 nm thick) was deposited over the sample through a sputtering evaporation system using the K550-Emitech sputter coater (Ashford, UK). The material was observed under a microscope (LEO 435 VP^®^), image methods (Detector = SE1, WD = 33 mm, voltage = 15 kV, Mag = 30 X).

### 2.9. Cell Viability Test

To evaluate the cell viability of the test specimens, a resazurin test was conducted. Three types of scaffolds were used: scaffold 1 (labyrinthine trabeculate, more delicate, with a rough surface); scaffold 2 (surface of trabeculae with smooth curvatures and gaps between them); and scaffold 3 (cross-shaped trabecular surface). Briefly, 7 mg of resazurin powder and sodium salt (Sigma-Aldrich-R7017-5G) was diluted in 50 mL of 1% PBS. Eleven measurements were tested: (1) culture medium only, (2) resazurin only, (3) culture medium + resazurin, (4) scaffold 1 + goat mesenchymal cells + resazurin, (4.1) scaffold 2 + goat mesenchymal cells + resazurin, (4.2) scaffold 3 + goat mesenchymal cells + resazurin, (5) goat mesenchymal cells + resazurin, (6) scaffold 1 + murine fibroblast cells + resazurin, (6.1) scaffold 2 + murine fibroblast cells + resazurin, (6.2) scaffold 3 + murine fibroblast cells + resazurin, and (7) murine fibroblast cells + resazurin. The test specimens were cultivated and placed in 24-well plates (KASVI-K12-024) with goat mesenchymal stem cells (MSCs) (2 × 10^3^/well) and 3T3 fibroblast cells (2 × 10^3^/well). The MSCs were supplemented with 1 mL of resazurin + 1 mL of Alpha-MEM-LGCBio medium, and the 3T3 cells were supplemented with 1 mL of DMEM (LGC Bio, Cotia, São Paulo, Brazil) medium. For analysis, 200 μL solution samples were collected on culture days 1, 4, 7, 10, and 18. The collected samples were stored in a refrigerator until the day of analysis (day 18). The solutions were distributed on a 96 well culture plate (KASVI-K12-096) and examined in a spectrophotometer (uQuant, Bio-Tek Instruments, INC., Winooski, VT, USA) at a wavelength of 540 nm. The data obtained were used to create proliferation graphs.

### 2.10. Statistical Analysis

Cell viability was assessed using ANOVA and a two-tailed unpaired Student’s *t* test for post hoc comparisons between the test pieces and the control, as well as between the two cell types over a period of 18 days. Statistical analysis was conducted using the GraphPad Prism software version 7.00 (GraphPad Software, San Diego, CA, USA). Data are expressed as mean ± SD. A *p*-value < 0.05 was considered statistically significant.

## 3. Results

### 3.1. Characterization of Resomer and Nanocomposites by FTIR Spectroscopy

**Figure 3:**
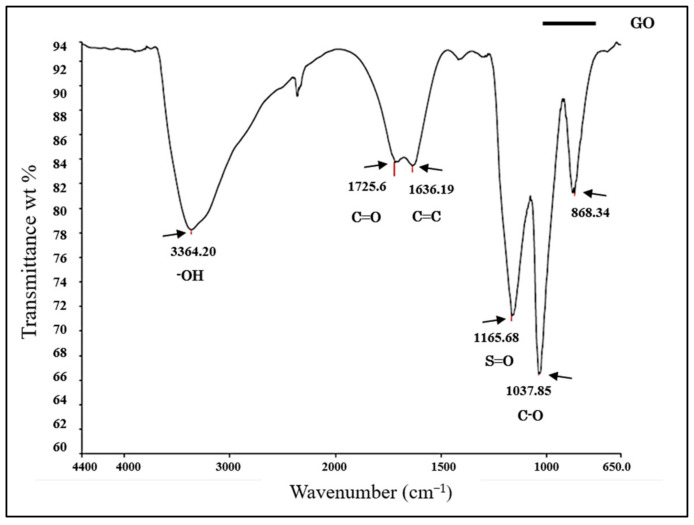
Fourier-transform infrared (FTIR) spectrum of graphene oxide.

The physicochemical composition of the resomer (PLLA) and GO/PLLA nanocomposites was assessed using the FTIR-ATR spectroscopy. The band spectrum observed in Figure 3 enabled us to identify the functional groups in each sample. For GO, the bands within the wavenumber range of 3600–3400 cm^−^^1^ corresponded to hydroxyl groups (OH), which include alcohols and carboxylic acids [37,38,39]. The band at 1725.6 cm^−^^1^ was attributed to the stretching of the C=O bond, indicating the presence of carboxylic acids [38]. At 1636.19 cm^−^^1^, the aromatic C=C bond was observed [40,41]. The band at 1165.68 cm^−^^1^ was associated with the S=O bond, which may be present due to the presence of sulfonic acids in the sample [37]. The vibrations observed at 1037.85 cm^−^^1^ were correlated with alcohols or phenols (C-O) [39]. Lastly, 868.34 cm^−^^1^ was correlated with the characteristics of a para substituted aromatic ring [39].

**Figure 4:**
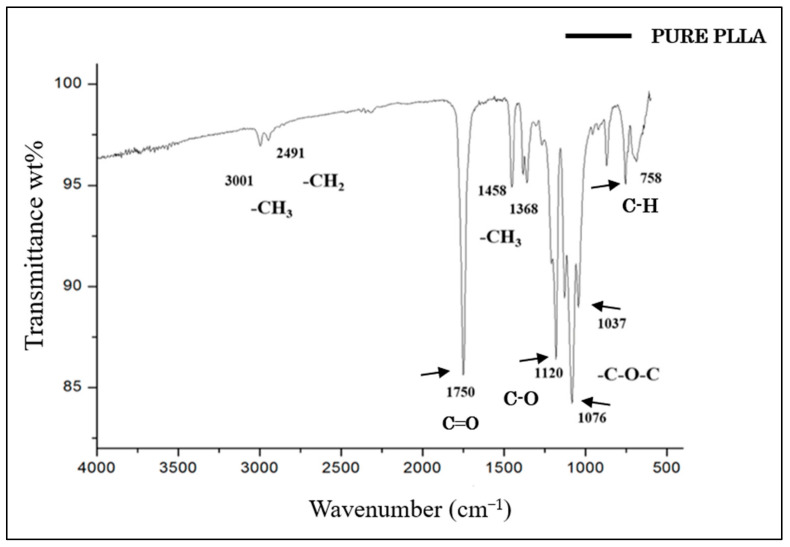
FTIR spectrum of pure PLLA.

The FTIR spectrum of PLLA showed no intense band in the range of 3500–3000 cm^−^^1^, which corresponded to the stretching of the OH group [42]. The most intense peak at 1750 cm^−^^1^ represented the stretching of the C=O group, which is related to carbonyls [42,43,44]. The band at 1458 cm^−^^1^ represented the asymmetric deformation of CH_3_ [43,44]. At 1368 cm^−^^1^, there was a band attributed to the CH deformation, which included symmetric and asymmetric vibrations, such as the symmetric deformation of CH_3_ [44]. At 1120 cm^−^^1^, the stretching of C-O was evident, while at 1076 cm^−^^1^ and 1043 cm^−^^1^, the elongation of C-O-C and C-CH_3_, respectively, was observed [42,43,44,45]. Finally, the band at 758 cm^−^^1^ represented the elongation and deformation of C-H, characteristic of the crystalline phase [45] (Figure 4).

**Figure 5:**
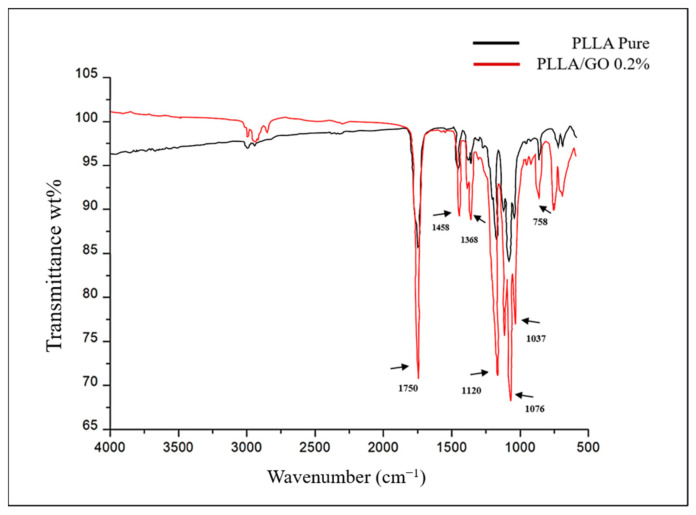
FTIR spectrum of pure poly-L-lactic acid (PLLA) and graphene oxide/poly-L-lactic acid (GO/PLLA).

The spectrum of the GO/PLLA bands exhibited a striking similarity to the peaks of the pure PLLA bands and showed few characteristics of the GO spectra, for example, the 1037 cm^−1^ and 1725 cm^−1^ bands, indicating that GO was likely present beneath the surface of the PLLA (Figure 5).

### 3.2. Characterization of the Resomer and Nanocomposites by Raman Spectroscopy

The thermal and chemical reductions used to produce GO generated defects in the grid [46]. These defects modified and determined the physical and chemical properties of graphene-based materials. In view of this, Raman spectroscopy is a widely used technique for studying these properties. 

**Figure 6:**
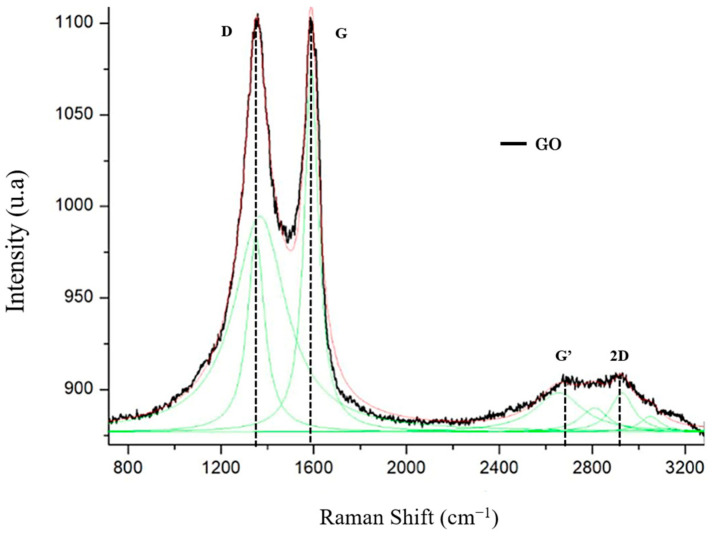
Raman spectrum of graphene oxide (GO).

In the GO spectroscopy, a distinct peak was observed at ≈1350 cm^−^^1^. This peak, denoted as the D band, was associated with structural defects in graphene [37]. Additionally, there was a distinct peak at approximately 1585 cm^−^^1^, denoted by the G band, which was related to the sp^2^ carbon atoms. The peak between 2600 and 2800 cm^−^^1^ is present in carbonaceous materials with sp^2^ hybridization (denoted by the G’ band). Lastly, the peaks at approximately 2800 and 3000 cm^−^^1^ (denoted as the 2D band) were correlated with the development of the graphene structure (Figure 6).

**Figure 7:**
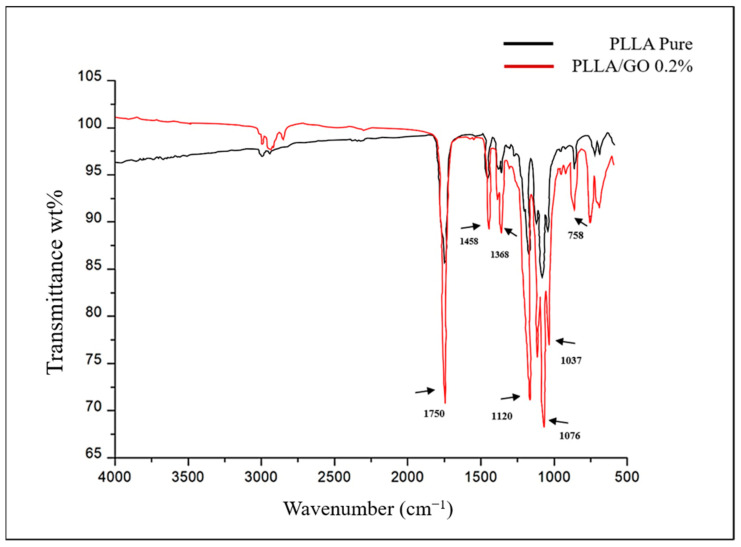
Raman spectrum of pure poly-L-lactic acid (PLLA) and its association with graphene oxide (GO).

The Raman spectrum of pure PLLA showed 10 distinct peaks (Figure 7). The first peak, observed at approximately 1041 cm^−^^1^, was attributed to the stretching mode (vC-CH_3_). The peak at ≈1091 cm^−^^1^ was related to the symmetric vibrations of the v(COC)s mode, while the peak at 1128 cm^−^^1^ was associated with the asymmetric band of r(CH_3_)as. The bands at approximately 1180 cm^−^^1^ and 1217 cm^−^^1^ were attributed to the C-O-C stretching modes of the ester groups, specifically as the asymmetric v(COC)as bands. Medium-intensity bands appeared at 1293 cm^−^^1^ and were mainly attributed to the bending vibrations of the methyl groups (δCH). The component of the δ1CH band coupled to the δ1CH mode was found in the spectrum at 1360 cm^−^^1^. The symmetric [δ(CH_3_)s] and asymmetric [δ(CH_3_)as] bending modes of the methyl groups were observed in the Raman spectrum at ~1389 cm^−^^1^ and ~1452 cm^−^^1^, respectively. Finally, the band at ~1768 cm^−^^1^ was correlated with the vC=O stretching. The spectrum of the GO/PLLA nanocomposites showed similar peaks.

### 3.3. Sterility and Cell Adhesion of Test Specimens and Critical Defect Scaffolds

**Figure 8:**
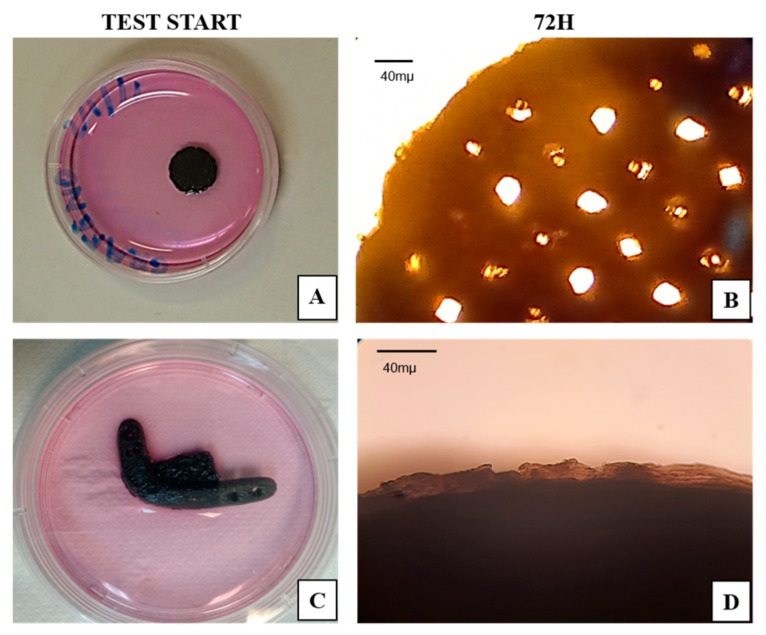
Sterility test. (**A**) Test piece at the beginning of testing; (**B**) test piece after 72 h; (**C**) critical defect scaffold at the beginning of testing; (**D**) critical defect scaffold after 72 h. Scale bar = 40 µm.

The sterility of the test specimens and the critical defect scaffolds were assessed by their contact with the α-MEM (LGC Bio) culture medium for 72 h. The non-contamination of the medium was indicated by its unchanged color (Figure 8).

**Figure 9:**
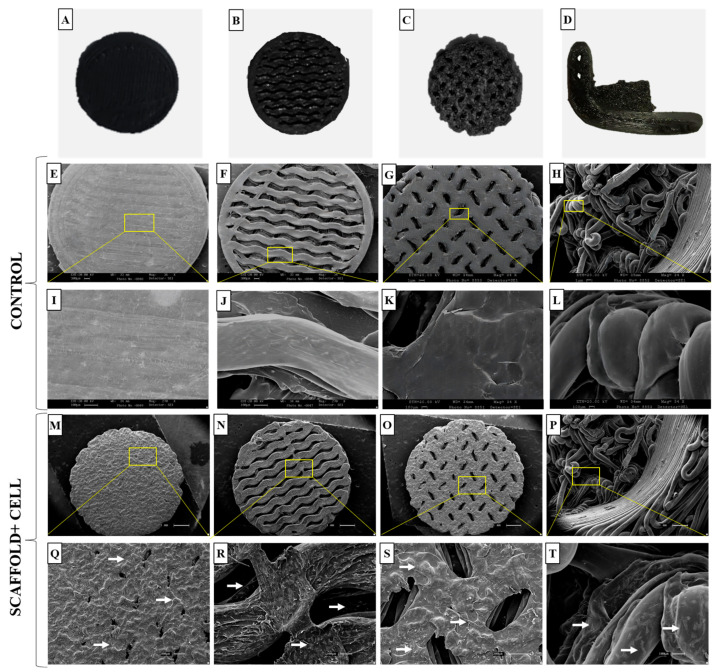
Macroscopic and ultrastructural images of test specimens and critical defect scaffold containing goat mesenchymal cells. (**A**–**C**) Different types of trabeculae and test specimens; (**D**) critical defect scaffold; (**E**–**G**) scanning electron microscopy (SEM) images of the test specimens’ control; (**H**) SEM images of the critical defect scaffold control, scale bar = 1 µm; (**I**–**L**) surface of the test specimens and critical defect scaffolds without cells, scale bar= 100 µm; (**M**–**O**) SEM images of the test specimens; (**P**) SEM images of the critical defect scaffold, scale bar= 1 µm; (**Q**–**T**) note the cells (white arrows) on the surface of the test specimens and in the inner layers of the critical defect scaffold with larger trabeculae, scale bar = 300 µm.

After conducting the sterility test, the cell adhesion capacity of these materials was evaluated by culturing goat mesenchymal cells for 7 days and examining them using SEM. Figure 9A–C show the different types of printed trabeculae, while Figure 9D shows the critical defect scaffold. SEM revealed the surface of the materials without cells (Figure 9E–L), when compared to the materials with cells (Figure 9M–T). It is possible to observe the difference between them, and how the cells adhered and grew throughout the nanocomposite structure homogeneously, regardless of the trabeculae. Still, it was possible to observe that the test specimens with different trabecular structure allowed for cell proliferation in the inner layers, such as the critical defect scaffold (Figure 9R,S,T). These pores are important so that the cells can expand, form connections, and fill the spaces inside the scaffold, imitating what occurs in the inner layer of bone tissue (bone trabeculae) by forming a three-dimensional grid that interconnects. 

### 3.4. Cell Viability

**Figure 10:**
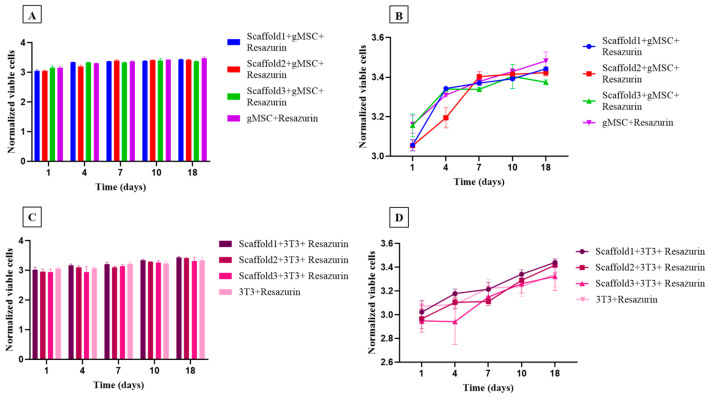
Viability and proliferation assessment showing the number of goat mesenchymal cells (*n* = 6) and the number of 3T3 fibroblast cells (*n* = 6) cultured in control medium (culture medium + resazurin) and in three different test specimens after 1, 4, 7, 10, and 18 days. (**A**,**B**) Trend and bar graph of goat mesenchymal cells; (**C**,**D**) trend and bar graph of fibroblast cells. Each bar represents the means of sextuplicate ± SD (*n* = 36), (*p*< 0.05).

The cytotoxicity of the nanocomposites was assessed using the resazurin assay after 1, 4, 7, 10, and 18 days (Figure 10). A similar growth pattern was observed for both goat mesenchymal stem cells (Figure 10A,B) and fibroblast cells (Figure 10C,D), with no statistical difference between them. During the 18-day experimentation period, the cells remained viable, and no differences were observed between scaffolds 1, 2, and 3, demonstrating excellent cytocompatibility of all the test specimens. From day 4 to day 7, a growth in cell proliferation was observed in both types of cells used. This growth continued linearly from day 7 to day 18, as shown in the trend graphs (Figure 10B,D).

## 4. Discussion

In this study, GO was combined with poly-L-lactic acid to create a nanocomposite that was used to produce filaments through extrusion. This approach enhanced the mechanical and biological properties of the polymer resulting in a printable material capable of generating customized scaffolds.

For use in BTE, these biomaterials must possess a rigid structure capable of serving as a matrix to support various cellular processes, including adhesion, viability, proliferation, and differentiation, thereby facilitating bone regeneration [47,48]. These biomaterials are expected to be particularly promising in biomedical engineering. They can serve as permanent implants, typically using nonbiodegradable materials (e.g., metals or ceramics), and as temporary scaffolds for tissue engineering, where biodegradable materials are preferred [49,50]. 

One of the main challenges of this study was to optimize the development of a scaffold that fulfilled the biological (viability, differentiation, and cell proliferation), since the trabecular bone structure is complex [51]. To meet these requirements, the structure must possess greater mechanical resistance with minimal use of materials while also allowing nutrients to permeate the cells. Triply periodic minimal (TPM) surfaces are independent, non-self-intersecting, and lightweight structures suitable for auditory manufacturing [52,53,54,55]. TPM surfaces can be adjusted to match the host’s tissue through the density/mechanical resistance ratio, similar to gyroid and Fischer–Koch structures [34,56]. In this study, the Fischer–Koch structure was used due to its closer resemblance to bone trabeculae, showing promising results [56,57]. 

Few studies have explored bone regeneration in the laboratory using this model due to the lack of algorithms to model and slice this topology for the use in low-cost biomaterial printers. In vitro studies have shown good permeability, cellular adherence, and viability inside test specimens, demonstrating that the architecture of TPM surfaces is promising for scaffold printing in bone tissue engineering [58,59].

Another aspect that must be considered when designing printable scaffolds is permeability, as it is related to the level of porosity and exhibits substantial variability depending on the chosen architecture. The chosen architecture is essential for achieving an optimal permeability/mechanical resistance ratio. Asbai-Goudan and Davar Ali [54,60] compared the permeability of printed structures using an analytical model adaptable to cell size and porosity and demonstrated a correlation between computational simulations and mechanical tests. This is important because the architecture of the tissue or material directly influences cellular reconstitution, especially cellular adhesion and proliferation, even if these parameters were not evaluated in this study.

Among the other analyses performed in this study, the FTIR characterization of GO and PLLA exhibited a pattern similar to that found by other authors [45,61,62,63]. However, they did not evaluate the association between these two biomaterials. In the present study, the infrared spectrum of GO/PLLA at 0.2% demonstrated that the position of its absorption peak (1750 cm^−^^1^ to PLLA) remained unaltered across the corresponding wavenumbers, indicating that these biomaterials did not change during the extrusion process. The Raman spectra for GO and PLLA presented peaks similar to those described in the literature [64]. The band intensity ratios were associated with an increase in the number and/or size of the atomic aggregates, suggesting that new graphitic materials were formed, thus showing the efficiency of the process [38]. Furthermore, the comparison between PLLA and GO/PLLA 0.2% also exhibited the same peak pattern but with different intensities. This difference between the spectral patterns is excellent, indicating that no chemical reaction occurred between the components of the different nanocomposites. This conclusion is supported by the fact that all identified peaks belonged to pure materials [65,66].

Raman spectroscopy was used in this study because it is a technique for analyzing structural changes and composition at the molecular level. It overcomes the limitations of conventional assays and provides information about the identification of toxic chemical products that may be present in the nanocomposites [67].

To explore the biomedical applications of GO/PLLA nanocomposites in bone tissue engineering (BTE), it was essential to investigate their biological behavior through a biocompatibility assay using goat mesenchymal stem cells (gMSCs). Cell adhesion is important because it directly influences cell proliferation and bone tissue formation [68]. Both GO and PLLA have been thoroughly investigated for biocompatibility [69,70,71,72]. As expected, our results indicated optimal adhesion of gMSCs to the trabeculae of the different composites, presenting a long shape similar to that of fibroblasts, as described in previous studies [73,74]. During cell seeding, the filament arrangement did not influence the effectiveness of the cell culture. Contrary to the observations by Yilgor et al. [75], pore size did not affect cell migration into the scaffold.

Biocompatibility was assessed through cell viability using a resazurin assay with murine 3T3 fibroblast cells and gMSCs. Our results demonstrate a gradual increase over the 18-day testing period, indicating that the nanocomposites enabled cellular anchoring and growth. This increase can be attributed to the presence of GO on the PLLA surface, which may improve cell proliferation and metabolic activity [71].

Moreover, various studies have reported that the GO contributes to the increase in cell viability [76,77]. Similarly, PLLA polymers have shown promising results when applied to bone scaffolds, as they increase the proliferation of various cell types [78,79]. In contrast, other studies have reported that the cytotoxicity of these materials depends on the cell type and assay used [80,81].

Continued investigation of the interactions between materials and cells, as observed in this study, may lead to the development of new materials that yield even more promising results in terms of cell viability. Future studies must evaluate printed nanocomposites in complex environments using animal models to analyze the behavior, degradation, and efficacy of the scaffold in a biological context closer to humans.

## 5. Conclusions

The aim of this study was to determine the feasibility of a printed scaffold focusing on the biomimetization of the bone structure and cell response to the printed construct. For that, GO/PLLA nanocomposites were characterized using FTIR spectroscopy, Raman spectroscopy, and SEM. Our results were consistent with those reported in the literature. When evaluated in vitro, the nanocomposites exhibited biocompatibility with gMSC cells during a 7-day culture period. Moreover, the nanocomposites enabled both murine 3T3 fibroblast cells and gMSCs to remain viable in the resazurin assay. Based on the data obtained here, the Fischer–Koch model presented satisfactory results and can thus be used in studies aimed at various medical applications, including bone tissue engineering and implants.

## Data Availability

Data sharing not applicable.
